# Sex Distributions in Non-*ABCA4* Autosomal Macular Dystrophies

**DOI:** 10.1167/iovs.65.5.9

**Published:** 2024-05-03

**Authors:** Amit V. Mishra, Sandra Vermeirsch, Siying Lin, Maria P. Martin-Gutierrez, Mark Simcoe, Nikolas Pontikos, Elena Schiff, Thales A. C. de Guimarães, Pirro G. Hysi, Michel Michaelides, Gavin Arno, Andrew R. Webster, Omar A. Mahroo

**Affiliations:** 1 Genetics Service, Moorfields Eye Hospital NHS Foundation Trust, London, United Kingdom; 2 UCL Institute of Ophthalmology, University College London, London, United Kingdom; 3 Section of Ophthalmology, King's College London, St. Thomas’ Hospital Campus, London, United Kingdom; 4 North East Thames Regional Genetics Service, Great Ormond Street Institute of Child Health, London, United Kingdom; 5 Department of Physiology, Development and Neuroscience, University of Cambridge, Cambridge, United Kingdom

**Keywords:** retina, macular dystrophies, macular degeneration, hereditary

## Abstract

**Purpose:**

We sought to explore whether sex imbalances are discernible in several autosomally inherited macular dystrophies.

**Methods:**

We searched the electronic patient records of our large inherited retinal disease cohort, quantifying numbers of males and females with the more common (non-*ABCA4*) inherited macular dystrophies (associated with *BEST1*, *EFEMP1*, *PROM1*, *PRPH2*, *RP1L1*, and *TIMP3*). *BEST1* cases were subdivided into typical autosomal dominant and recessive disease. For *PRPH2*, only patients with variants at codons 172 or 142 were included. Recessive *PROM1* and recessive *RP1L1* cases were excluded because these variants give a more widespread or peripheral degeneration. The proportion of females was calculated for each condition; two-tailed binomial testing was performed. Where a significant imbalance was found, previously published cohorts were also explored.

**Results:**

Of 325 patients included, numbers for *BEST1*, *EFEMP1*, *PROM1*, *PRPH2*, *RP1L1*, and *TIMP3* were 152, 35, 30, 50, 14, and 44, respectively. For autosomal dominant Best disease (*n* = 115), there were fewer females (38%; 95% confidence interval [CI], 29–48%; *P* = 0.015). For *EFEMP1*-associated disease (*n* = 35), there were significantly more females (77%; 95% CI, 60%–90%; *P* = 0.0019). No significant imbalances were seen for the other genes. When pooling our cohort with previous large dominant Best disease cohorts, the proportion of females was 37% (95% CI, 31%–43%; *P* = 1.2 × 10^−5^). Pooling previously published *EFEMP1*-cases with ours yielded an overall female proportion of 62% (95% CI, 54%–69%; *P* = 0.0023).

**Conclusions:**

This exploratory study found significant sex imbalances in two autosomal macular dystrophies, suggesting that sex could be a modifier. Our findings invite replication in further cohorts and the investigation of potential mechanisms.

Sex differences in disease cohorts have increasingly been identified in the epidemiologic literature. This also applies to the field of ophthalmology, with reports of sex differences in many retinal diseases. Increased propensity to affect one or the other sex in Coats disease and subtypes of macular telangiectasia are well-established. Retinal tears and detachments have been found to have a significant male preponderance,^1^^,^^2^ whereas macular holes,^3^ vitreomacular traction, and lamellar macular holes are found more frequently in females.^4^ Late AMD is also seen more commonly in females.^5^

In monogenic retinal diseases, an obvious imbalance will occur in X-linked conditions, whereas autosomally inherited retinal diseases are presumed to affect both sexes equally. However, a recent study by Runhart et al.^6^ found a female preponderance in patients with mild *ABCA4* alleles causing Stargardt disease. The imbalance was not present in the group of patients with nonmild alleles.^6^ A later study also explored this question, but did not find a sex imbalance in patients with mild *ABCA4* alleles.^7^ A large meta-analysis is currently ongoing to seek a more definitive answer in the case of *ABCA4* retinopathy.

We sought to investigate this question for other autosomal macular dystrophies in our large, genetically characterized inherited retinal disease cohort at Moorfields Eye Hospital.^8^^,^^9^ The genes explored in the present study are *BEST1*, *EFEMP1*, *PROM1*, *PRPH2*, *RP1L1*, and *TIMP3* (all identified as being the most commonly associated with autosomal macular dystrophies in our cohort, after *ABCA4*).^8^ If any sex imbalance was a result of potential confounding factors such as having more females in the population or behavioral differences in seeking medical review, we would expect to see a similar effect across all autosomal dystrophies. We divided cases with *BEST1*-associated disease into autosomal dominant and recessive bestrophinopathies. With regard to *PRPH2*, phenotypes can vary widely,^10^ so we restricted our analysis to missense changes at two codons (142 and 172), which more frequently cause macular dystrophy in our cohort. Other variants in *PRPH2* are known to cause a wide variety of retinal phenotypes, including classic retinitis pigmentosa.^10^ Similarly, for *PROM1*- and *RP1L1*-associated disease, autosomal recessive cases were excluded, because these entail a more widespread or peripheral retinopathy.

## Methods

This cross-sectional study adhered to the tenets of the Declaration of Helsinki. Patients underwent genetic testing using a variety of methods and research ethics approval was from Moorfields Eye Hospital and the Northwest London Research Ethics Committee. A retrospective search of the electronic patient record was done at a single large center (Moorfields Eye Hospital). All patients referred for suspected inherited retinal disease were examined by an experienced retinal specialist. All patients have a detailed clinical history and ophthalmic examination and imaging, typically including SD-OCT and short-wavelength fundus autofluorescence. Genetic testing was performed using a variety of methods over the years: the majority of testing was by sequencing of gene panels and, more recently, by whole genome sequencing. The genetic testing strategy, and demographic makeup, of our inherited retinal disease cohort has been described in greater detail previously.^8^^,^^9^

The genes for the present study were chosen by examining the top 40 genes implicated in inherited retinal disease as identified in our previous study,^8^ and then excluding *ABCA4* as well as those genes that were X linked or that were not predominantly associated with a macular dystrophy. This yielded the following genes (in descending order of numbers of families affected): *PRPH2*, *BEST1*, *PROM1*, *TIMP3*, *EFEMP1*, and *RP1L1*. Because the data from our previous study derived from a search conducted more than 4 years ago, and a large number of patients with inherited retinal disease have been genetically diagnosed since that time, an updated search of the electronic patient record was conducted in relation to these genes.^9^

Patients with diseases associated with *BEST1* variants were divided into those with autosomal dominant Best disease or recessive bestrophinopathy. Any patients with nonmaculopathy phenotypes (such as retinitis pigmentosa or retina-wide degenerations) were excluded. Because *PRPH2* can be associated with a diverse range of phenotypes, including retinitis pigmentosa, only those patients with pathogenic variants affecting codon 172 or codon 142 were included (because these variants more consistently give rise to macular dystrophies in our cohort). For *PROM1*-associated disease, only those with autosomal dominant disease were included, because this tends to give rise to a predominant macular dystrophy phenotype (although peripheral involvement can occur). Similarly, for *RP1L1*-associated disease, only those with autosomal dominant disease (where the phenotype is a maculopathy, also termed occult macular dystrophy) were included.

The proportion of females (with 95% confidence intervals [95% CI]) was calculated in each condition. Also, a two-tailed binomial test was performed to examine whether the proportion of either sex was significantly different from 50%. Although a *P* value of less than 0.05 is usually taken as nominally significant, we were conducting seven separate tests. A strict Bonferroni correction yielded a corrected *P* value threshold of 0.0071.

## Results

**Figure 1.**
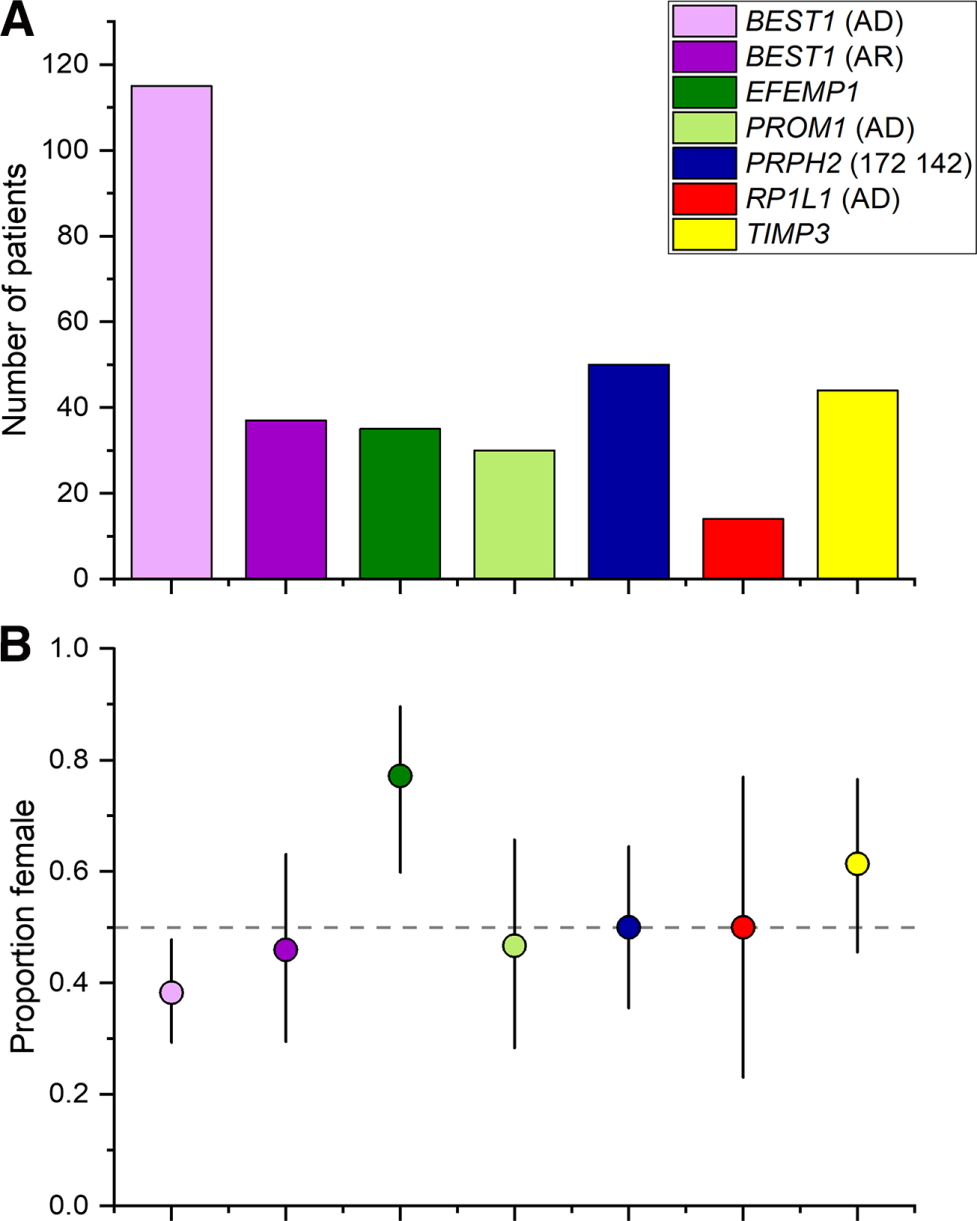
Numbers of patients and proportion of females with each monogenic condition. (**A**) Numbers of patients in each group. (**B**) Proportion of females with 95% CI denoted by error bars. The horizontal dashed line shows 50%.

A total of 325 patients were included: numbers with variants in *BEST1*, *EFEMP1*, *PROM1*, *PRPH2*, *RP1L1*, and *TIMP3* were 152, 35, 30, 50, 14, and 44, respectively. One patient with dominant *BEST1*-associated disease, but a retinitis pigmentosa phenotype, was excluded. Table gives the number of males and females for each condition and the significance level for the binomial test. The top of Figure 1 shows overall numbers and the bottom shows the proportions of females. The error bars represent 95% CIs and these do not cross 50% for two of the conditions.

**Table.** Numbers and Sex Distributions for Inherited Macular Dystrophies With Results of Two-tailed Binomial Testing

	No. of Patients				
Genetic Subgroup	Total	Males	Females	Female % (95% CI)	*P* Value for Imbalance
*BEST1*					
Dominant	115	71	44	38.3 (29.4–47.8)	0.015
Recessive	37	20	17	46.0 (29.5–63.1)	0.743
*EFEMP1*	35	8	27	77.1 (59.9–89.6)	0.0019^*^
*PROM1* (Dominant)	30	16	14	46.7 (28.3–65.7)	0.467
*PRPH2* (172 142)	50	25	25	50.0 (35.5–64.5)	1
*RP1L1* (Dominant)	14	7	7	50.0 (23.0–77.0)	1
*TIMP3*	44	17	27	61.4 (45.5–76.5)	0.174

* *P* < 0.0071 denotes significance.

**Figure 2.**
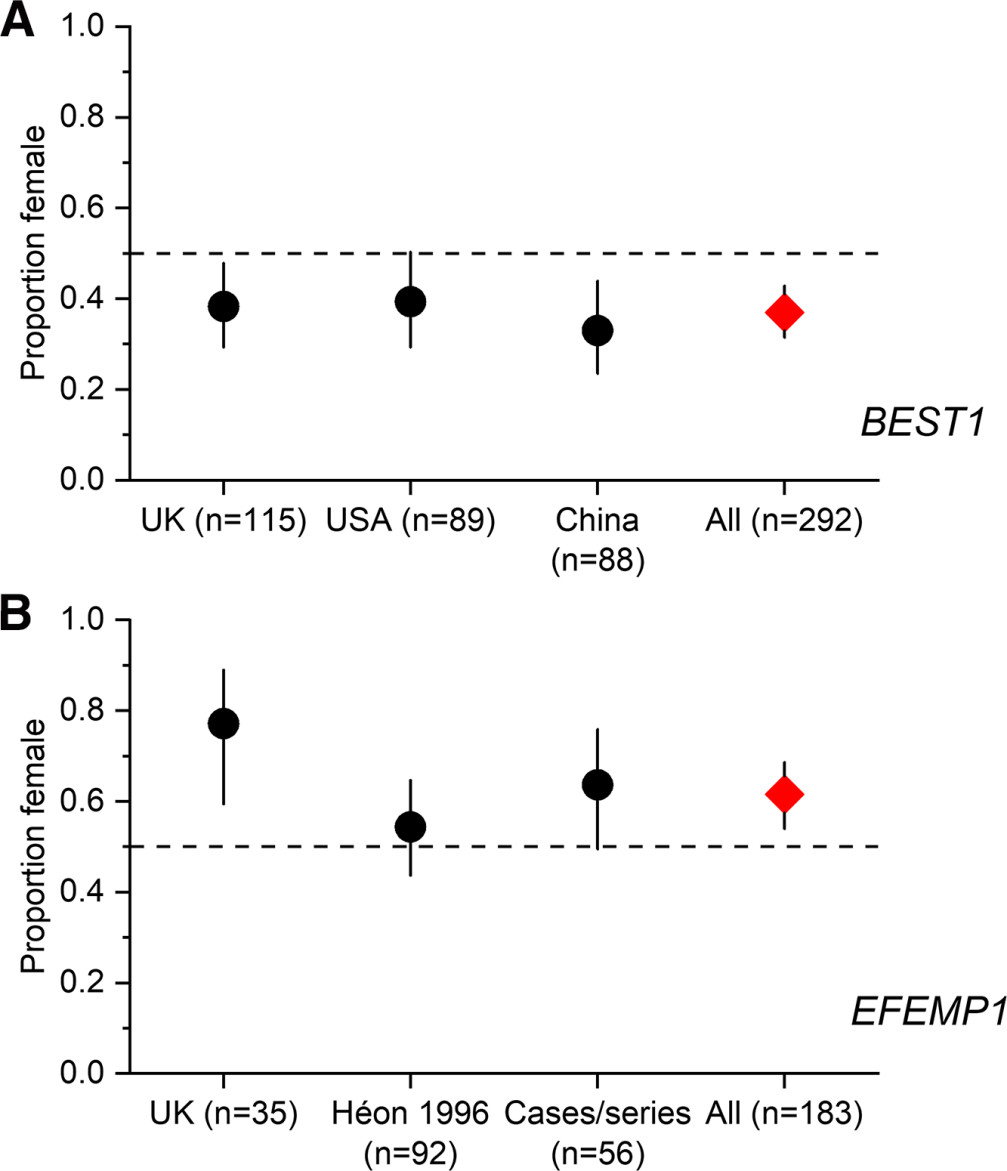
Proportions of females in patients with autosomal dominant Best disease and *EFEMP1*-associated disease. Proportion of females with 95% CI denoted by error bars. The horizontal dashed line shows 50%. (**A**) Proportions in Best disease. Black circles relate to cohorts from the UK (present study), United States,^11^ and China.^12^ The *red diamond* depicts the proportion when all three cohorts are pooled. (**B**) Proportions in *EFEMP1*-associated dominant drusen. *Black circles* plot proportions from the UK (present study), from an early large study of five families,^14^ and from many cases pooled from many subsequent reports.^15^^–^^29^ The *red diamond* plots the proportion when all these cases are pooled.

In autosomal dominant *BEST1* disease (*n* = 115), there was a sex imbalance toward males (proportion of females, 38%; 95% CI, 29–48%; *P* = 0.015), although this difference did not reach Bonferroni significance. No significant sex difference was seen in autosomal recessive *BEST1* disease, but the overall numbers were smaller (*n* = 37). A significant female preponderance was seen in patients with *EFEMP1*-associated disease (77%; 95% CI, 60%–90%; *P* = 0.0019). No significant sex difference was observed in the remaining genes (*PROM1*, *PRPH2*, *RP1L1*, and *TIMP3*).

### Pooling With Previously Published Best and EFEMP1-Associated Disease Cohorts

We found two previously published large cohorts of patients with dominant Best disease from the United States (*n* = 89)^11^ and China (*n* = 88).^12^ In the former study, there were 54 men and 35 women; in the latter study, there were 59 men and 28 women, yielding proportions of females of 39% and 33%, respectively, similar to our proportion of 38%. Figure 2 (top) plots these proportions with CIs. When we pooled our Best disease patients with the two other cohorts^11^^,^^12^ (*n* = 292), the overall proportion of females was 37% (95% CI, 31%–43%; *P* = 1.2 × 10^−5^), indicating a significant preponderance of males.

For *EFEMP1*, the gene discovery paper^13^ included a large number of patients, but did not report their sex. An earlier paper^14^ that pointed to the relevant chromosomal locus did show pedigrees for five families (92 affected individuals), from which numbers of affected males and females could be extracted (these were 40 and 52, respectively). Since then, several publications have reported additional cases^15^^–^^29^: these cases were reviewed (avoiding mutual overlap or overlap with the cohort in the current study); of 55 reported patients, 20 were males and 35 were females. The bottom of Figure 2 plots proportions with CIs. When these cases were pooled with our cohort, the overall proportion of females was 62% (95% CI, 54%–69%; *P* = 0.0023).

## Discussion

A sex imbalance was identified in autosomal dominant *BEST1* disease (male preponderance) and *EFEMP1*-related disease (female preponderance). For dominant Best disease, the initial *P* value (*P* = 0.015) exceeded the threshold of significance when corrected for multiple testing, but the finding of a similar imbalance in other cohorts from different countries is highly suggestive. Indeed, when we pooled our patients with Best disease with the two other cohorts^11^^,^^12^ (*n* = 292), the statistical significance increased markedly (*P* = 1.2 × 10^−5^). For *EFEMP1*, our initial *P* value was significant (*P* = 0.0019). When looking at previously published cases, a larger cohort also had more affected females than males, but here the calculated CI overlaps 50% (Fig. 2B). However, when pooling those cases with subsequent reports and with our cohort, the overall imbalance was significant (*P* = 0.0023).

We did not find significant imbalances for the other genes. In the literature relating to those genes, sex imbalances have not usually been explored, and are not generally apparent or consistent. For *PROM1* and *TIMP3*, many published series are relatively small. For *PRPH2*, the mutational spectrum in a large cohort (103 families from Spain) was published recently.^30^ In that study, sex was given for 181 patients: 107 were female. Applying binomial testing yields a *P* value of 0.017. Of 103 patients from that cohort who had a macular dystrophy phenotype, 60 were female (*P* = 0.11). In our study, we focused on variants at two particular codons. Taking our autosomal dominant *PRPH2*-associated cohort overall (regardless of variant), no significant imbalance was seen: 94 of 186 patients were female (*P* = 0.88). For *RP1L1*-associated occult macular dystrophy, a recent publication reported findings in 51 East Asian patients^31^: 21 were female (yielding a *P* value of 0.26). A previously reported, mainly German, cohort of 42 patients^32^ had 23 females (*P* = 0.58). Luoma-Overstreet et al.^33^ reviewed cases of occult macular dystrophy published before their report, and described similar numbers of males (*n* = 143) and females (*n* = 141), although they pointed out that these numbers included both *RP1L1*-associated asymptomatic cases and cases of occult macular dystrophy without a confirmed *RP1L1* variant.

The mechanisms for sex imbalances in some autosomally inherited macular dystrophies are unclear. Although we do not propose that pathogenic alleles are more likely to be inherited by one sex, their manifestation as symptomatic disease might be modified by sex (i.e., penetrance or disease expressivity might differ by sex), as has been suggested in the case of mild alleles in *ABCA4*-associated disease.^6^ Population studies have shown differences in macular thickness between the sexes, persisting even after adjustment for other factors.^34^^–^^36^ Segmented outer retinal layer thicknesses have been shown to be lower in females.^35^ Also, messenger RNAs for estrogen, progesterone, and androgen receptors have been identified in retinal cells^37^^,^^38^; it is plausible that sex hormones play a role in macular physiology, and act as a modifying factor in macular disease. Future studies can examine whether markers of disease severity, including age of onset, might differ by sex in autosomal dystrophies.

Our study does have limitations. Because inherited macular dystrophies are rare, our sample size is small, which limits the overall power, particularly for the rarer conditions. Our main cohort was derived from a large single institution and so might not be generalizable to other populations. Selection bias could have influenced our findings. It is possible that apparent sex differences can emerge from sex differences in the population and also differences in healthcare seeking behavior.^39^^,^^40^ However, such factors might be less likely to explain the findings of the present study, given that we observed sex imbalances in some conditions and not in others, and also in opposite directions in our patient cohort for two different conditions.

In a previous study,^2^ the hypothesis was explored that the well-reported preponderance of males in retinal detachment datasets (even after excluding trauma-related etiologies)^1^ might be related to female patients being more likely to seek medical attention after noticing symptoms relating to a retinal tear. If females were more likely than males to present to an eye specialist and undergo retinopexy, then this could theoretically contribute to an apparent excess of males in retinal detachment data. It would be predicted then that retinopexy data would show a greater proportion of female patients. However, when this hypothesis was tested, a significant preponderance of males was observed in the retinopexy data,^2^ suggesting that the eyes of male patients are more likely to develop both retinal tears and detachments. Hence, caution is warranted before attributing sex imbalances to differences in behavior without further investigation.

This cross-sectional study identifies sex as a potential modifier in some autosomal macular dystrophies, and thus should be given consideration in future research. Future elucidation of the mechanisms by which modifiers act may yield avenues for investigation of novel therapies. Our study also invites further work in larger, multicenter patient cohorts and in other autosomal retinal dystrophies.
